# Head-to-Head Comparison of 8 Plasma Amyloid-β 42/40 Assays in Alzheimer Disease

**DOI:** 10.1001/jamaneurol.2021.3180

**Published:** 2021-09-20

**Authors:** Shorena Janelidze, Charlotte E. Teunissen, Henrik Zetterberg, José Antonio Allué, Leticia Sarasa, Udo Eichenlaub, Tobias Bittner, Vitaliy Ovod, Inge M. W. Verberk, Kenji Toba, Akinori Nakamura, Randall J. Bateman, Kaj Blennow, Oskar Hansson

**Affiliations:** 1 Clinical Memory Research Unit, Department of Clinical Sciences Malmö, Lund University, Lund, Sweden; 2 Neurochemistry Laboratory, Department of Clinical Chemistry, Amsterdam Neuroscience, Vrije Universiteit Amsterdam, Amsterdam University Medical Center, Amsterdam, the Netherlands; 3 Institute of Neuroscience & Physiology, Department of Psychiatry and Neurochemistry, the Sahlgrenska Academy at the University of Gothenburg, Mölndal, Sweden; 4 Clinical Neurochemistry Laboratory, Sahlgrenska University Hospital, Mölndal, Sweden; 5 Department of Neurodegenerative Disease, University College London Institute of Neurology, London, United Kingdom; 6 United Kingdom Dementia Research Institute at University College London, London, United Kingdom; 7 Mass Spectrometry Laboratory, Araclon Biotech, Zaragoza, Spain; 8 Roche Diagnostics, Penzberg, Germany; 9 F. Hoffmann-La Roche, Basel, Switzerland; 10 Department of Neurology, Washington University School of Medicine, St Louis, Missouri; 11 National Center for Geriatrics and Gerontology, Obu, Aichi, Japan; 12 Tokyo Metropolitan Institute of Gerontology, Tokyo, Japan; 13 Center for Development of Advanced Medicine for Dementia, National Center for Geriatrics and Gerontology, Obu, Aichi, Japan; 14 Department of Psychiatry and Neurochemistry, Institute of Neuroscience & Physiology, the Sahlgrenska Academy at the University of Gothenburg, Mölndal, Sweden; 15 Memory Clinic, Skåne University Hospital, Malmö, Sweden

## Abstract

**Importance:**

Blood-based tests for brain amyloid-β (Aβ) pathology are needed for widespread implementation of Alzheimer disease (AD) biomarkers in clinical care and to facilitate patient screening and monitoring of treatment responses in clinical trials.

**Objective:**

To compare the performance of plasma Aβ42/40 measured using 8 different Aβ assays when detecting abnormal brain Aβ status in patients with early AD.

**Design, Setting, and Participants:**

This study included 182 cognitively unimpaired participants and 104 patients with mild cognitive impairment from the BioFINDER cohort who were enrolled at 3 different hospitals in Sweden and underwent Aβ positron emission tomography (PET) imaging and cerebrospinal fluid (CSF) and plasma collection from 2010 to 2014. Plasma Aβ42/40 was measured using an immunoprecipitation-coupled mass spectrometry developed at Washington University (IP-MS-WashU), antibody-free liquid chromatography MS developed by Araclon (LC-MS-Arc), and immunoassays from Roche Diagnostics (IA-Elc); Euroimmun (IA-EI); and Amsterdam University Medical Center, ADx Neurosciences, and Quanterix (IA-N4PE). Plasma Aβ42/40 was also measured using an IP-MS–based method from Shimadzu in 200 participants (IP-MS-Shim) and an IP-MS–based method from the University of Gothenburg (IP-MS-UGOT) and another immunoassay from Quanterix (IA-Quan) among 227 participants. For validation, 122 participants (51 cognitively normal, 51 with mild cognitive impairment, and 20 with AD dementia) were included from the Alzheimer Disease Neuroimaging Initiative who underwent Aβ-PET and plasma Aβ assessments using IP-MS-WashU, IP-MS-Shim, IP-MS-UGOT, IA-Elc, IA-N4PE, and IA-Quan assays.

**Main Outcomes and Measures:**

Discriminative accuracy of plasma Aβ42/40 quantified using 8 different assays for abnormal CSF Aβ42/40 and Aβ-PET status.

**Results:**

A total of 408 participants were included in this study. In the BioFINDER cohort, the mean (SD) age was 71.6 (5.6) years and 49.3% of the cohort were women. When identifying participants with abnormal CSF Aβ42/40 in the whole cohort, plasma IP-MS-WashU Aβ42/40 showed significantly higher accuracy (area under the receiver operating characteristic curve [AUC], 0.86; 95% CI, 0.81-0.90) than LC-MS-Arc Aβ42/40, IA-Elc Aβ42/40, IA-EI Aβ42/40, and IA-N4PE Aβ42/40 (AUC range, 0.69-0.78; *P* < .05). Plasma IP-MS-WashU Aβ42/40 performed significantly better than IP-MS-UGOT Aβ42/40 and IA-Quan Aβ42/40 (AUC, 0.84 vs 0.68 and 0.64, respectively; *P* < .001), while there was no difference in the AUCs between IP-MS-WashU Aβ42/40 and IP-MS-Shim Aβ42/40 (0.87 vs 0.83; *P* = .16) in the 2 subcohorts where these biomarkers were available. The results were similar when using Aβ-PET as outcome. Plasma IPMS-WashU Aβ42/40 and IPMS-Shim Aβ42/40 showed highest coefficients for correlations with CSF Aβ42/40 (*r* range, 0.56-0.65). The BioFINDER results were replicated in the Alzheimer Disease Neuroimaging Initiative cohort (mean [SD] age, 72.4 [5.4] years; 43.4% women), where the IP-MS-WashU assay performed significantly better than the IP-MS-UGOT, IA-Elc, IA-N4PE, and IA-Quan assays but not the IP-MS-Shim assay.

**Conclusions and Relevance:**

The results from 2 independent cohorts indicate that certain MS-based methods performed better than most of the immunoassays for plasma Aβ42/40 when detecting brain Aβ pathology.

## Key Points

**Question:**

How well does plasma amyloid-β 42/40 (Aβ42/40), measured using 8 different assays, detect brain Aβ pathology in the early stages of Alzheimer disease?

**Findings:**

In this study, including 408 participants from 2 independent cohorts (BioFINDER and Alzheimer Disease Neuroimaging Initiative), plasma Aβ42/40 quantified using certain mass spectrometry–based methods showed better discriminative accuracy than immunoassays when identifying individuals with abnormal intracerebral Aβ status according to cerebrospinal fluid Aβ42/40 levels and Aβ positron emission tomography.

**Meaning:**

Certain mass spectrometry–based plasma tests might have sufficient performance to detect brain Aβ pathology in Alzheimer disease.

## Teaser

This cross-sectional study compares the performance of plasma amyloid-β 42/40 (Aβ42/40) measured using 8 different Aβ assays and methods in detecting abnormal brain Aβ status in patients with early Alzheimer disease.

## Introduction

Blood tests for detecting amyloid-β (Aβ) pathology in Alzheimer disease (AD) would be a major advancement for biomarker implementation in clinical care and highly useful in drug trials.^1^ Reliable measurements of Aβ in blood proved challenging^2^ until the development of advanced mass spectrometry and immunodetection methods. In 2016, plasma Aβ42/40 assessed using an ultrasensitive Simoa immunoassay was shown to detect abnormal cerebrospinal fluid (CSF) Aβ or Aβ-positron emission tomography (PET) status with moderate accuracy.^3^ Plasma Aβ42/40 determined with high-precision immunoprecipitation-coupled mass spectrometry (IP-MS) was later reported to correlate with Aβ-PET and identify with high precision individuals with abnormal brain Aβ burden or those at high risk of future conversion to Aβ-PET positivity.^4,5,6^ More recent articles have suggested that Aβ42/40 quantified using ultrasensitive and fully automated immunoassay platforms could predict Aβ-PET status (especially when combined with *APOE* genotype) with accuracy approaching that of MS-based Aβ42/40 measures.^7,8^ However, the varying performance of the different Aβ assays and platforms across the studies could be at least in part owing to the differences in the cohort characteristics (eg, sample size, included diagnostic groups, and outcome measures) and preanalytical sample handling. To minimize these biases, we performed a head-to-head comparison of 8 Aβ assays in the same cohort of individuals with early AD from the Swedish BioFINDER study. We assessed how well plasma Aβ42/40 measured using different assays could discriminate abnormal from normal CSF Aβ42/40 or Aβ-PET status. Finally, we replicated findings from BioFINDER using data from the Alzheimer Disease Neuroimaging Initiative (ADNI).

## Methods

### Participants

The study included 286 individuals from the prospective Swedish BioFINDER-1 (NCT03174938) cohort recruited between 2010 and 2014. Among the BioFINDER participants, 182 were cognitively unimpaired elderly individuals and 104 had mild cognitive impairment (MCI). For study design and recruitment procedures, see the eMethods in the Supplement. The BioFINDER study was approved by the Regional Ethics Committee in Lund, Sweden. All participants provided written informed consent. Data were analyzed from March 2021 to July 2021.

For validation, we selected 120 participants (51 cognitively unimpaired, 51 with MCI, and 20 with AD dementia) recruited between 2005 and 2013 from ADNI who had plasma Aβ assessments. Data were obtained from the ADNI database.^9^ ADNI was launched in 2003 as a public-private partnership led by Principal Investigator Michael W. Weiner, MD. Ethical approval was given by the local ethical committees of all involved sites. Data were analyzed from June 2021 to July 2021.

### Plasma and CSF Analysis

All BioFINDER study participants underwent measurements of plasma concentrations of Aβ42 and Aβ40 using the IP-MS–based method developed at Washington University, St Louis, Missouri (IP-MS-WashU), the antibody-free liquid chromatography–MS developed by Araclon Biotech, Zaragoza, Spain (LC-MS-Arc), Elecsys immunoassays from Roche Diagnostics, Penzberg, Germany (IA-Elc), immunoassays from Euroimmun, Lübeck, Germany (IA-EI), and N4PE Simoa immunoassays (IA-N4PE) developed by Amsterdam University Medical Center, Amsterdam, the Netherlands, and ADx Neurosciences, Ghent, Belgium, and commercially available from Quanterix, Billerica, Massachusetts, in the specific laboratories.^4,5,6,7,8,10,11,12^ In subcohorts of study participants, plasma samples were analyzed using the IP-MS–based method developed by Shimadzu, Kyoto, Japan (IP-MS-Shim; n = 200; subcohort 1), as well as the IP-MS–based methods developed at the University of Gothenburg, Gothenburg, Sweden (IP-MS-UGOT), and another Simoa immunoassay from Quanterix (IA-Quan; n = 227; subcohort 2).^3,4,11^ Aβ42 and Aβ40 levels in CSF were determined with Elecsys CSF immunoassays. We included all participants from BioFINDER who underwent [^18^F]flutemetamol PET imaging (n = 416) with plasma samples available at the time of analysis except that the samples were randomly selected for the IP-MS-Shim, IP-MS-UGOT, and IA-Quan assays. In ADNI, plasma concentrations of Aβ42 and Aβ40 were quantified using IP-MS-WashU, IP-MS-Shim, IP-MS-UGOT, IA-Elc, IA-N4PE, and IA-Quan. All participants in ADNI who had plasma Aβ and Aβ-PET assessments were included. Further details of blood and CSF collection and analysis are described in the eMethods and eTables 1 and 2 in the Supplement.

### Aβ-PET Imaging

In BioFINDER, Aβ imaging was performed using [^18^F]flutemetamol PET 90 to 110 minutes postinjection, as described in the eMethods in the Supplement. Standardized uptake value ratio was defined as the uptake in a global neocortical target region of interest with the cerebellar cortex as reference region.^13^ In ADNI, Aβ imaging was performed using [^18^F]florbetapir PET 50 to 70 minutes postinjection using a global neocortical target region of interest with the whole cerebellum as reference region.^14,15^

### Statistical Analysis

SPSS version 22 (IBM) was used for statistical analysis. Correlations between biomarkers were assessed with the Spearman test. Differences between the groups were tested using Mann-Whitney *U* test or Fisher exact test. Unadjusted 2-sided *P* values <0.05 were considered statistically significant. Discrimination accuracies of biomarkers were determined with logistic regression models and receiver operating characteristic curve analysis. Area under the receiver operating characteristic curve (AUC) of 2 receiver operating characteristic curves were compared with DeLong test with adjustment for multiple comparisons using a false discovery rate of 5%. In BioFINDER, CSF Aβ42/40 was used as the outcome in the main analysis. We also performed a sensitivity analysis with Aβ-PET and CSF Aβ42/40 measured with the Euroimmun assay as outcomes to ensure that the results were not biased by the use of the same antibodies in the CSF and plasma for the Elecsys Aβ42/40 assays. In ADNI, CSF Aβ42 and Aβ40 measures at the time of plasma collection were only available in a small group of participants, and therefore we used Aβ PET as the outcome. CSF Aβ42/40 and Aβ-PET data were binarized using previously described cutoffs (CSF Aβ42/40 Elecsys, 0.059; CSF Aβ42/40 Euroimmun, 0.091; Aβ-PET BioFINDER, 1.42; ADNI, 1.11).^7,13,14,15,16^

## Results

### Participants in BioFINDER

Of the 286 participants without dementia in BioFINDER, 141 (49.3%) were women, and the mean (SD) age was 71.6 (5.6) years. The baseline demographic and clinical characteristics of the whole cohort as well as the 2 subcohorts with IP-MS-Shim Aβ42/40 or IA-Quan Aβ42/40 data available are summarized in Table 1 and eTables 3 and 4 in the Supplement, respectively. For all tested assays, plasma Aβ42 and Aβ42/40 were lower in individuals who were Aβ positive compared with those who were Aβ negative whereas there were no differences in the levels of Aβ40 (Table 1; eTables 3, 4, and 5 in the Supplement).

**Table 1.** Characteristics of Study Participants in BioFINDER

Characteristic	Median (IQR)		*P* value^b^
	Aβ negative (n = 168)^a^	Aβ positive (n = 118)^a^	
Diagnosis, CU/MCI, No.	127/41	55/63	<.001
Age, y	71.0 (67.0-75.0)	74.0 (70.0-77.0)	.001
Female, No. (%)	90 (53.6)	51 (43.2)	.93
Duration of education, y^c^	12.0 (9.0-14.0)	11.0 (9.0-13.0)	.91
MMSE	29.0 (28.0-30.0)	28.0 (26.0-29.0)	<.001
APOE ε4 positivity, No. (%)^d^	35 (21.0)	77 (65.3)	<.001
Aβ-PET, [^18^F]flutemetamol SUVR	1.19 (1.12-1.28)	1.86 (1.57-2.15)	<.001
CSF Aβ42/40	0.093 (0.079-0.102)	0.041 (0.034-0.049)	<.001
Plasma Aβ42/40			
IP-MS-WashU	0.132 (0.126-0.139)	0.122 (0.117-0.126)	<.001
LC-MS-Arc	0.322 (0.298-0.346)	0.288 (0.266-0.304)	<.001
IA-Elc	0.068 (0.064-0.072)	0.062 (0.058-0.065)	<.001
IA-EI	0.179 (0.162-0.199)	0.162 (0.146-0.174)	<.001
IA-N4PE	0.135 (0.119-0.147)	0.119 (0.105-0.132)	<.001

Abbreviations: Aβ, amyloid-β; CSF, cerebrospinal fluid; CU, cognitively unimpaired; IA-EI, immunoassay from Euroimmun; IA-Elc, Elecsys immunoassay from Roche Diagnostics; IA-N4PE, N4PE Simoa immunoassay from Quanterix; IP-MS-WashU, immunoprecipitation-coupled mass spectrometry method developed at Washington University; IQR, interquartile range; LC-MS-Arc, antibody-free liquid chromatography-mass spectrometry method developed by Araclon; MCI, mild cognitive impairment; MMSE, Mini-Mental State Examination; PET positron emission tomography; SUVR, standardized uptake value ratio.

^a^ Aβ status was defined using the CSF Aβ42/40 cutoff (0.059) derived from mixture modeling as previously described.^7^

^b^ Differences between the groups were tested using Mann-Whitney *U* test and Fisher exact test (diagnosis, sex, and *APOE*).

^c^ Education is missing for 2 study participants.

^d^ *APOE* ε4 is missing for 1 study participant.

### Prediction of CSF Aβ Status Using Different Plasma Aβ Assays in BioFINDER

When identifying individuals with abnormal CSF Aβ42/40 in the whole cohort (Figure, A; Table 2), plasma IP-MS-WashU Aβ42/40 had significantly better discriminative accuracy (AUC, 0.86; 95% CI, 0.81-0.90) than plasma LC-MS-Arc Aβ42/40 (AUC, 0.78; 95% CI, 0.72-0.83; *P* < .01), IA-Elc Aβ42/40 (AUC, 0.78; 95% CI, 0.73-0.83; *P* < .01), IA-EI Aβ42/40 (AUC, 0.70; 95% CI, 0.64-0.76; *P* < .001), and IA-N4PE Aβ42/40 (AUC, 0.69; 95% CI, 0.63-0.75; *P* < .001).

**Figure.**
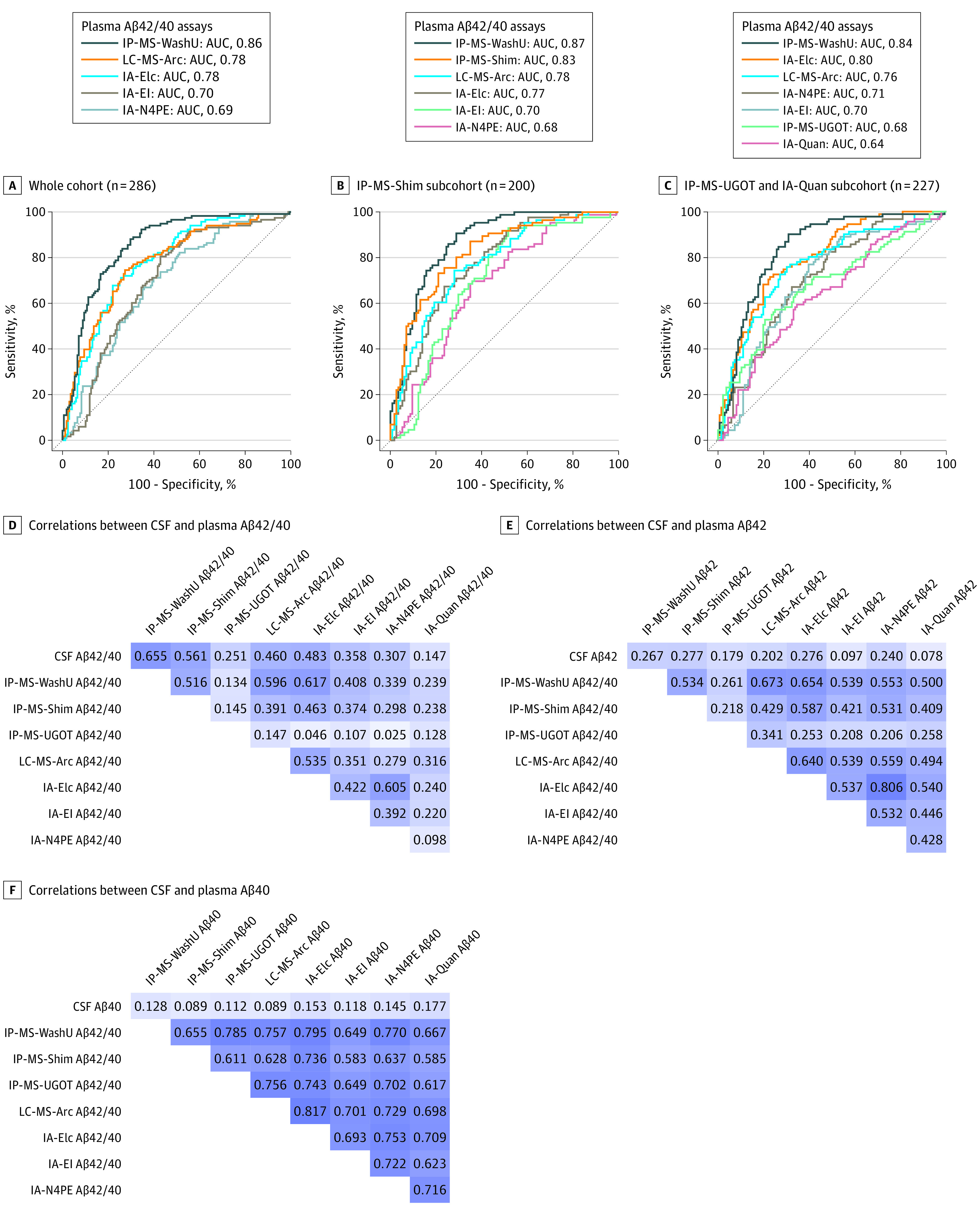
Receiver Operating Characteristic (ROC) Analysis for Abnormal Cerebrospinal Fluid (CSF) Amyloid-β42/40 (Aβ42/40) and Correlations Between CSF and Plasma Aβ A, ROC curve analysis for differentiating participants with abnormal CSF Aβ42/40 from those with normal CSF Aβ42/40 (cutoff, 0.0597) in the whole cohort. B, ROC curve analysis in the subcohorts where IPMS-Shim Aβ42/40 was available. C, ROC curve analysis in the subcohorts where IPMS-UGOT and IA-Quan Aβ42/40 were available. D, Spearman correlations between plasma and CSF Aβ42/40 in a subcohort (n = 155) individuals where all plasma samples were analyzed using all 8 assays. E, Spearman correlations between plasma and CSF Aβ42 in a subcohort (n = 155) where all plasma samples were analyzed using all 8 assays. F, Spearman correlations between plasma and CSF Aβ40 in a subcohort (n = 155) where all plasma samples were analyzed using all 8 assays.

**Table 2.** Receiver Operating Curve (ROC) Analysis for Abnormal Cerebrospinal Fluid (CSF) Amyloid-β42/40 (Aβ42/40) and Aβ–Positron Emission Tomography (PET) Status in BioFINDER^a^

Plasma Aβ42/40 Assay	AUC (95% CI)^b^	
	CSF Aβ42/40	Aβ-PET
Entire cohort		
Aβ+, No.	118	110
Aβ−, No.	168	176
IP-MS-WashU	0.855 (0.810-0.899)	0.833 (0.787-0.879)
IA-Elc	0.778 (0.725-0.832)^c^	0.727 (0.669-0.784)^d^
LC-MS-Arc	0.776 (0.721-0.830)^c^	0.753 (0.696-0.811)^c^
IA-EI	0.697 (0.635-0.758)^d^	0.672 (0.609-0.735)^d^
IA-N4PE	0.687 (0.626-0.748)^d^	0.655 (0.591-0.719)^d^
Subcohort with IP-MS-Shim Aβ42/40^e^		
Aβ+, No.	86	86
Aβ−, No.	114	114
IP-MS-WashU	0.872 (0.824-0.920)	0.872 (0.824-0.920)
IP-MS-Shim	0.825 (0.767-0.882)	0.825 (0.767-0.882)
LC-MS-Arc	0.775 (0.711-0.839)^c^	0.775 (0.711-0.839)^c^
IA-Elc	0.773 (0.709-0.837)^c^	0.773 (0.709-0.837)^c^
IA-EI	0.704 (0.631-0.777)^d^	0.704 (0.631-0.777)^d^
IA-N4PE	0.679 (0.605-0.753)^d^	0.679 (0.605-0.753)^d^
Subcohort with IP-MS-UGOT and IA-Quan Aβ42/40		
Aβ+, No.	91	86
Aβ−, No.	136	141
IP-MS-WashU	0.838 (0.785-0.891)	0.814 (0.760-0.868)
IA-Elc	0.795 (0.738-0.853)	0.728 (0.663-0.793)^c^
LC-MS-Arc	0.763 (0.700-0.827)^f^	0.742 (0.676-0.809)^f^
IA-N4PE	0.706 (0.639-0.773)^c^	0.649 (0.577-0.721)^d^
IA-EI	0.697 (0.628-0.767)^d^	0.667 (0.596-0.738)^d^
IP-MS-UGOT	0.678 (0.605-0.750)^d^	0.632 (0.557-0.707)^d^
IA-Quan	0.636 (0.563-0.709)^d^	0.600 (0.525-0.675)^d^

Abbreviations: AUC, area under the curve; IA-EI, immunoassay from Euroimmun; IA-Elc, Elecsys immunoassay from Roche Diagnostics; IA-N4PE, N4PE Simoa immunoassay from Quanterix; IA-Quan, Simoa immunoassay from Quanterix; IP-MS-Shim, immunoprecipitation coupled mass spectrometry method developed by Shimadzu; IP-MS-WashU, immunoprecipitation-coupled mass spectrometry method developed at Washington University; IP-MS-UGOT, immunoprecipitation-coupled mass spectrometry method developed at the University of Gothenburg; LC-MS-Arc, antibody-free liquid chromatography-mass spectrometry method developed by Araclon.

^a^ CSF Aβ42/40 and Aβ-PET data were binarized using previously described cutoffs (0.059 and 1.42, respectively).^7,13^

^b^ AUC of 2 ROC curves were compared with DeLong test.

^c^ *P* < .01, compared with IP-MS-WashU Aβ42/40.

^d^ *P* < .001, compared with IP-MS-WashU Aβ42/40.

^e^ In this subcohort, CSF Aβ42/40 and Aβ-PET concordance was 100%.

^f^ *P* < .05 compared with Aβ42/40IP-MS-WashU.

In the 2 subcohorts of participants where IP-MS-Shim Aβ42/40 or IP-MS-UGOT Aβ42/40 and IA-Quan Aβ42/40 were also available, IP-MS-WashU Aβ42/40 showed higher discriminative accuracy for CSF Aβ42/40 status than IP-MS-UGOT Aβ42/40 (AUC, 0.84; 95% CI, 0.79-0.89 vs AUC, 0.68; 95% CI, 0.61-0.75; *P* < .001) and IA-Quan Aβ42/40 (AUC, 0.84; 95% CI, 0.79-0.89 vs AUC, 0.64; 95% CI, 0.56-0.71; *P* < .001), while the difference in AUCs between IP-MS-WashU Aβ42/40 and IP-MS-Shim Aβ42/40 was not significant (AUC, 0.87; 95% CI, 0.82-0.92 vs AUC, 0.83; 95% CI, 0.77-0.88; *P* = .16) (Figure, B and C, Table 2).

For comparison, one of the most promising plasma biomarkers of AD, p-tau217,^10,17^ distinguished 117 individuals with abnormal CSF Aβ42/40 from 168 individuals with normal CSF Aβ42/40 with an AUC of 0.79 (95% CI, 0.74-0.84), which was numerically lower but not significantly different from the AUC of IP-MS-WashU Aβ42/40 (0.86; 95% CI, 0.81-0.90; unadjusted *P* = .06).

### Sensitivity Analyses in BioFINDER

The results were similar when using CSF Aβ42/40 analyzed with the Euroimmun immunoassay instead of the Elecsys immunoassay as the reference standard (eTable 6 in the Supplement). Further, the overall results were very similar when using Aβ-PET as the outcome, with most assays showing numerically lower AUCs compared with AUCs for CSF Aβ42/40 as the outcome (Table 2).

### Correlations Between Plasma and CSF Aβ in BioFINDER

Spearman coefficients were highest for correlations of CSF Aβ42/40 with plasma IP-MS-WashU Aβ42/40 (*r*, 0.65; *P* < .001), followed by IP-MS-Shim Aβ42/40 (*r*, 0.56; *P* < .001), IA-Elc Aβ42/40 (*r*, 0.48; *P* < .001), LC-MS-Arc Aβ42/40 (*r*, 0.46; *P* < .001), IA-EI Aβ42/40 (*r*, 0.36; *P* < .001), IA-N4PE Aβ42/40 (*r*, 0.31; *P* < .001), IP-MS-UGOT Aβ42/40 (r, 0.25, P = .002) and IA-Quan Aβ42/40 (*r*, 0.15; *P* = .06) (Figure, D). Further, there were correlations between plasma Aβ measured using different assays for both Aβ42 (*r* range; 0.21-0.81) and Aβ40 (*r* range, 0.58-0.82), but the coefficients were lower for correlations between plasma and CSF Aβ42 (*r* range, 0.08-0.28) and plasma and CSF Aβ40 (*r* range, 0.09-0.18) (Figure, E and F for all assays used in a subcohort of 155 participants; eFigure in the Supplement for the 5 assays used in the whole cohort).

### Combining Plasma Aβ With APOE ε4 in BioFINDER

Adding *APOE* ε4 status improved the accuracy of all Aβ42/40 measures (ΔAUC, 0.027-0.140; eTable 7 in the Supplement) with the AUCs of the 3 MS-based methods and IA-Elc Aβ42/40 consistently above 0.82 in the whole cohort and the 2 subcohorts in which AUCs differences between the 3 MS-based methods lost statistical significance.

### Validation in ADNI

Of 122 participants in ADNI, 53 (43.4%) were women, and the mean (SD) age was 72.4 (5.4) years. The baseline demographic and clinical characteristics are summarized in Table 3. For all 6 tested assays, plasma Aβ42/40 was lower in individuals who were Aβ positive compared with individuals who were Aβ negative whereas there were no differences in the levels of Aβ40 (Table 3; eTable 8 in the Supplement). Plasma Aβ42 concentrations were also lower in the Aβ-positive group than in Aβ-negative group for all assays except IA-Quan, which did not show significant differences between the groups (eTable 8 in the Supplement). In ADNI, for IP-MS-Shim, we used a previously described composite biomarker score because it identified abnormal Aβ-PET more accurately than Aβ42/40 in this cohort. Similar to the results in BioFINDER, we found that plasma IP-MS-WashU Aβ42/40 showed better performance (AUC, 0.85; 95% CI, 0.77-0.92) than plasma IP-MS-UGOT Aβ42/40 (AUC, 0.66; 95% CI, 0.57-0.76; *P* < .001), IA-Elc Aβ42/40 (AUC, 0.74; 95% CI, 0.65-0.83; *P* < .05), IA-N4PE Aβ42/40 (AUC, 0.69; 95% CI, 0.59-0.78; *P* < .01), and IA-Quan Aβ42/40 (AUC, 0.63; 95% CI, 0.53-0.73; *P* < .001) but not IP-MS-Shim composite biomarker score (AUC, 0.82; 95% CI, 0.75-0.89; *P* = .54) (Table 4).

**Table 3.** Characteristics of Study Participants in the Alzheimer Disease Neuroimaging Initiative

Characteristic	Median (IQR)		*P* value^b^
	Aβ negative (n = 63)^a^	Aβ positive (n = 59)^a^	
Diagnosis, CN/MCI/AD, No.	35/26//2	16/25/18	<.001
Age, y	70.7 (65.7-76.0)	74.2 (69.9-77.5)	.02
Female, No. (%)	28 (44.4)	25 (42.4)	.86
Duration of education, y	18.0 (15.0-19.0)	16.0 (13.0-18.0)	.24
MMSE	29.0 (28.0-30.0)	27.0 (23.0-29.0)	<.001
APOE ε4 positivity, No. (%)	18 (28.6)	32 (54.2)	.006
Aβ-PET, [^18^F]florbetapir SUVR	1.006 (0.960-1.037)	1.321 (1.235-1.470)	<.001
CSF Aβ42/40	NA	NA	NA
Plasma Aβ42/40			
IP-MS-WashU	0.132 (0.128-0.141)	0.122 (0.117-0.127)	<.001
IP-MS-Shim	0.040 (0.037-0.045)	0.037 (0.034-0.039)	<.001
IP-MS-UGOT	0.071 (0.061-0.089)	0.064 (0.052-0.073)	.002
IA-Elc	0.171 (0.154-0.182)	0.152 (0.141-0.164)	<.001
IA-N4PE	0.049 (0.042-0.054)	0.043 (0.039-0.047)	<.001
IA-Quan	0.040 (0.037-0.044)	0.037 (0.034-0.041)	.01

Abbreviations: Aβ, amyloid-β; AD, Alzheimer disease dementia; CSF, cerebrospinal fluid; CU, cognitively unimpaired; IA-Elc, Elecsys immunoassay from Roche Diagnostics; IA-N4PE, N4PE Simoa immunoassay from Quanterix; IA-Quan, Simoa immunoassay from Quanterix; IP-MS-Shim, immunoprecipitation coupled mass spectrometry method developed by Shimadzu; IP-MS-UGOT, immunoprecipitation-coupled mass spectrometry method developed at the University of Gothenburg; IP-MS-WashU, immunoprecipitation-coupled mass spectrometry method developed at Washington University; IQR, interquartile range; MCI, mild cognitive impairment; MMSE, Mini-Mental State Examination; PET, positron emission tomography; SUVR, standardized uptake value ratio.

^a^ Aβ status was defined using a previously described Aβ-PET cutoff (1.11).^14,15^

^b^ Differences between the groups were tested using Mann-Whitney *U* test, χ^2^ (diagnosis), or Fisher exact test (sex and *APOE*).

**Table 4.** Receiver Operating Curve (ROC) Analysis for Abnormal Aβ-PET in the Alzheimer Disease Neuroimaging Initiative^a^

Plasma assay	Aβ-PET, AUC (95% CI)^b^
Aβ+, No.	59
Aβ−, No.	63
Aβ42/40 IP-MS-WashU	0.845 (0.772-0.917)
Composite IP-MS-Shim	0.821 (0.747-0.895)
Aβ42/40 IA-Elc	0.740 (0.651-0.829)^c^
Aβ42/40 IA-N4PE	0.685 (0.590-0.781)^d^
Aβ42/40 IP-MS-UGOT	0.662 (0.565-0.758)^e^
Aβ42/40 IA-Quan	0.634 (0.534-0.734)^e^

Abbreviations: Aβ, amyloid-β; AUC, area under the curve; IA-Elc, Elecsys immunoassay from Roche Diagnostics; IA-N4PE, N4PE Simoa immunoassay from Quanterix; IA-Quan, Simoa immunoassay from Quanterix; IP-MS-Shim, immunoprecipitation coupled mass spectrometry method developed by Shimadzu; IP-MS-UGOT, immunoprecipitation-coupled mass spectrometry method developed at the University of Gothenburg; IP-MS-WashU, immunoprecipitation-coupled mass spectrometry method developed at Washington University; ROC, receiver operating characteristic; PET, positron emission tomography.

^a^ In ADNI, CSF Aβ42 and Aβ40 measures at the time of plasma collection were only available in a small group of participants, and therefore we used Aβ PET as the outcome.

^b^ AUCs of 2 ROC curves were compared with DeLong test. Aβ-PET data was binarized using a previously described threshold of 1.11. ^14,15^

^c^ *P* < .05, compared with IP-MS-WashU Aβ42/40.

^d^ *P* < .01, compared with IP-MS-WashU Aβ42/40.

^e^ *P* < .001, compared with Aβ42/40IP-MS-WashU.

## Discussion

In this cross-sectional study examining the performance of 8 plasma assays for quantification of Aβ42/40, we found that certain MS-based methods offered better precision than immunoassays for identifying individuals with early AD. In 2 independent cohorts, 2 IP-MS methods (IP-MS-WashU and IP-MS-Shim) had the highest discriminative accuracy for determining CSF Aβ42/40 and Aβ-PET status. In BioFINDER, Spearman coefficients were highest for correlations of CSF Aβ42/40 with IP-MS-WashU Aβ42/40 and IP-MS-Shim Aβ42/40 as well.

Aβ42/40 measured using IP-MS has previously shown high accuracy in detecting abnormal brain Aβ status in AD with AUCs ranging from 0.88 to 0.97,^4,5,6^ and the IP-MS blood test for Aβ developed by Washington University can now be used in clinical care in US. In the present study, the AUCs of both IP-MS–based methods were somewhat lower (0.82-0.87) than in other cohorts, highlighting that the impact of differences in cohort characteristics and sample handling is not negligible. Nevertheless, plasma Aβ42/40 quantified with the IP-MS-WashU approach showed significantly better performance than the immunoassays. These findings could be explained by high specificity of MS-based technologies in general, which is considered a substantial advantage over immunoassay, but also by differences in the antibody specificities and sample handling procedures. It is also possible that the Aβ IP-MS methods are less prone to matrix effects that can be especially pronounced in protein-rich and compositionally complex biological fluids such as blood.^18^ However, while MS is a powerful research tool, fully automated immunoassays or MS will probably be needed to provide global access to blood-based biomarkers for routine clinical use in primary care settings. Among the immunoassays, IA-Elc Aβ42/40 had the numerically highest AUC, most likely because Elecsys Aβ immunoassays are performed on a fully automated platform with very high analytical reliability and precision.^19^

### Limitations

This study has limitations. One limitation is that IP-MS-Shim Aβ42/40, IP-MS-UGOT Aβ42/40, and IA-Quan Aβ42/40 were not available in the whole cohort. Other limitations include the relatively small size of the Aβ-negative cognitively unimpaired group and that the assays were performed at different laboratories, possibly introducing some preanalytical variation. Future investigations should examine the performance of different Aβ methods separately in cognitively unimpaired participants and those with MCI.

## Conclusions

In conclusion, plasma Aβ42/40 determined using certain MS-based methods identified individuals with abnormal brain Aβ burden more accurately than immunoassay-based Aβ42/40 measures. These findings can help inform the future clinical use of blood tests for Aβ pathology in AD.

## Notes

Supplement. eMethods eFigure. Correlations between plasma and CSF Aβ in the whole cohort (n=286) eTable 1. Plasma Aβ42 and Aβ40 assays in BioFINDER eTable 2. Preanalytical sample handling in BioFINDER eTable 3. Characteristics of a subcohort of participants in BioFINDER where plasma Aβ was measured using IP-MS-Shim eTable 4. Characteristics of a subcohort of participants in BioFINDER where plasma Aβ was measured using IP-MS-UGOT and IA-Quan eTable 5. Plasma concentrations of Aβ42 and Aβ40 in BioFINDER eTable 6. ROC analysis for abnormal CSF Aβ42 and Aβ40EI status in BioFINDER eTable 7. ROC analysis of plasma Aβ42 and Aβ40 combined with APOE ε4 genotype for abnormal CSF Aβ42 and Aβ40 in BioFINDER eTable 8. Plasma concentrations of Aβ42 and Aβ40 in ADNI eReferences eAppendix. ADNI Investigators
