# Supplementary material for: Head-to-Head Comparison of 8 Plasma Amyloid-β 42/40 Assays in Alzheimer Disease
Source: JAMA Neurol. 2021 Sep 20;78(11):1–8. doi: 10.1001/jamaneurol.2021.3180 (PMC8453354; doi:10.1001/jamaneurol.2021.3180)
Supplement: Supplement. — eMethods eFigure. Correlations between plasma and CSF Aβ in the whole cohort (n=286) eTable 1. Plasma Aβ42 and Aβ40 assays in BioFINDER eTable 2. Preanalytical sample handling in BioFINDER eTable 3. Characteristics of a subcohort of participants in BioFINDER where plasma Aβ was measured using IP-MS-Shim eTable 4. Characteristics of a subcohort of participants in BioFINDER where plasma Aβ was measured using IP-MS-UGOT and IA-Quan eTable 5. Plasma concentrations of Aβ42 and Aβ40 in BioFINDER eTable 6. ROC analysis for abnormal CSF Aβ42 and Aβ40EI status in BioFINDER eTable 7. ROC analysis of plasma Aβ42 and Aβ40 combined with APOE ε4 genotype for abnormal CSF Aβ42 and Aβ40 in BioFINDER eTable 8. Plasma concentrations of Aβ42 and Aβ40 in ADNI eReferences eAppendix. ADNI Investigators [file jamaneurol-e213180-s001.pdf]

Supplemental Online Content

Janelidze S, Teunissen CE, Zetterberg H, et al. Head-to-head comparison of 8 plasma amyloid-β 42/40 assays in Alzheimer disease. *JAMA Neurol*. Published online September 20, 2021. doi:10.1001/jamaneurol.2021.3180

eMethods

eFigure. Correlations between plasma and CSF Aβ in the whole cohort (n=286)

eTable 1. Plasma Aβ42 and Aβ40 assays in BioFINDER

eTable 2. Preanalytical sample handling in BioFINDER

eTable 3. Characteristics of a subcohort of participants in BioFINDER where plasma Aβ was measured using IP-MS-Shim

eTable 4. Characteristics of a subcohort of participants in BioFINDER where plasma Aβ was measured using IP-MS-UGOT and IA-Quan

eTable 5. Plasma concentrations of Aβ42 and Aβ40 in BioFINDER

eTable 6. ROC analysis for abnormal CSF Aβ42 and Aβ40EI status in BioFINDER

eTable 7. ROC analysis of plasma Aβ42 and Aβ40 combined with APOE ε4 genotype for abnormal CSF Aβ42 and Aβ40 in BioFINDER

eTable 8. Plasma concentrations of Aβ42 and Aβ40 in ADNI

eReferences

eAppendix. ADNI Investigators

This supplemental material has been provided by the authors to give readers additional information about their work.

© 2021 Janelidze S et al. *JAMA Neurology.*

eMethods

BioFINDER

Participants

We included 286 participants from the prospective Swedish BioFINDER-1 (NCT03174938) study recruited between 2010 and 2014 at Skåne University Hospital and the Hospital of Ängelholm, Sweden. Cognitively healthy elderly individuals were randomly enrolled from a population-based community cohort study in Malmö, Sweden (Malmö Diet and Cancer Study). Patients with subjective cognitive decline (SCD) or mild cognitive impairment (MCI) were consecutively included after referral from the primary care to the memory clinics. The inclusion criteria for cognitively healthy elderly were 1) absence of cognitive symptoms as assessed by a physician with special interest in cognitive disorders, 2) age ≥60 years, 3) MMSE 28-30 points at screening visit, 4) did not fulfill the criteria for mild cognitive impairment (MCI) or any dementia disorder, and 5) fluency in Swedish. The exclusion criteria were 1) significant unstable systemic illness or organ failure, such as terminal cancer, that made it difficult to participate in the study, 2) current significant alcohol or substance misuse and 3) significant neurological or psychiatric illness. The inclusion criteria for patients with SCD or MCI (defined using criteria by Petersen[1]) were (1) referred to a participating memory clinic because of cognitive complaints, (2) age 60 to 80 years, (3) did not fulfill the criteria for any dementia disorder and (4) fluency in Swedish. The exclusion criteria were 1) significant unstable systemic illness or organ failure, such as terminal cancer, that made it difficult to participate in the study, 2) current significant alcohol or substance misuse and 3) cognitive impairment that without doubt could be explained by other specific non-neurodegenerative disorders, such as brain tumor or subdural hematoma. Patients were classified as SCD or MCI following neuropsychological assessment including a test battery evaluating verbal ability, episodic memory function, visuospatial construction ability, and attention and executive functions.[2] In accordance with the research framework by the National Institute on Aging-Alzheimer's Association study patients with SCD and cognitively healthy individuals were considered as cognitively unimpaired (CU).[3] In this study, we selected all participants from BioFINDER who underwent [^{18}F]flutemetamol PET imaging (n=416) with plasma samples available at the time of analysis except that the samples were randomly selected for the IP-MS-Shim, IP-MS-UGOT and IA-Quan assays.

Plasma and cerebrospinal fluid (CSF) sampling

Blood and CSF samples were collected in the morning during the same visit with participants non-fasting. Blood was collected in 6 EDTA-plasma tubes (Vacutainer® K2EDTA tube, BD Diagnostics) and centrifuged (2000g, +4°C) for 10 min. Following centrifugation, plasma from all 6 tubes was transferred into one 50ml polypropylene tubes tube, mixed and 1ml was aliquoted into 1.5ml polypropylene tubes and stored at -80°C within 30-60 min of collection.

In all study participants, plasma concentrations of Aβ42 and Aβ40 were measured using previously described IP-MS-based method developed at Washington University (IP-MS-WashU), antibody-free liquid chromatography-MS developed by Araclon (LC-MS-Arc) as well as Elecsys immunoassays from Roche Diagnostics International Ltd (IA-Elc), immunoassays from Euroimmun (IA-EI) and N4PE Simoa immunoassays from Quanterix (IA-N4PE).[4-7] In sub-cohorts of study participants, plasma samples were analyzed using IP-MS-based method developed by Shimadzu (IP-MS-Shim) and another Simoa immunoassay from Quanterix (IA-Quan).[8,9] Aβ42 and Aβ40 levels in CSF were determined with Elecsys and Euroimmun CSF immunoassays.

Plasma and CSF Aβ analysis

IP-MS-WashU

Plasma samples were spiked with ^{15}N-Aβ40 and ^{15}N-Aβ42 for use as analytical reference standards. Aβ42 and Aβ40 isoforms were immunoprecipitated using a monoclonal anti-Aβ mid-domain antibody (HJ5.1, anti-Aβ13-28). Liquid chromatography-tandem mass spectrometry (LC-MS/MS) and analysis of mass spectrometry data were performed as previously described.[4,10] Plasma samples were analyzed at the Department of Neurology, Washington University School of Medicine (St. Louis, MO, USA) between September 2019 and January 2020.

IP-MS-Shim

Plasma samples were with the SIL-Aβ1–38 peptide was used as internal standard for normalization of signals for all Aβ-related peptides in the mass spectrum. Aβ42 and Aβ40 isoforms were immunoprecipitated using a

© 2021 Janelidze S et al. *JAMA Neurology.*

monoclonal anti-Aβ 6E10 antibody. MALDI-TOF mass spectrometry and analysis of mass spectrometry data were performed as previously described.[9] Analysis of Aβ40 and Aβ42 failed in 37 plasma samples, there was no second aliquot to repeat the analysis and, therefore, these samples were excluded from the present study. Samples were analyzed at the Shimadzu Tchno-Research Inc. (Kyoto, Japan) between January and February 2019.

LC-MS-Arc

Plasma Aβ40 and Aβ42 concentrations were measured using an antibody-free liquid chromatography-differential mobility spectrometry-triple quadrupole mass spectrometric (HPLC-DMS-MS/MS) method.[11] The analytical platform was composed of a QTRAP 6500+ SelexION hybrid linear ion trap-triple quadrupole mass spectrometer, fitted with a differential mobility spectrometry interface and coupled to a M3 Micro LC system (all from Sciex, Framingham, MA, USA). Analytes were extracted directly from plasma; no immunoprecipitation procedure was followed. Intact Aβ40 and Aβ42 species were measured as no enzymatic digestion was performed. Analysis of Aβ40 and Aβ42 failed in 60 plasma samples due to an over-pressure in the chomatographic system, there was no second aliquot to repeat the analysis and, therefore, these samples were excluded from the present study. Samples were analyzed at Araclon Biotech Ltd. (Zaragoza, Spain) in November 2019.

IP-MS-UGOT

^{15}N-Aβ40 and ^{15}N-Aβ42 recombinant peptides were added to samples and Aβ peptides were immunoprecipitated with anti-amyloid-β antibodies 4G8 (epitope 17–27 in the amyloid-β sequence) and 6E10 (epitope 1–16, both antibodies from BioLegend). LC-MS/MS and analysis of mass spectrometry data were performed as previously described. [12] Analysis of Aβ40 and Aβ42 failed in 14 plasma samples, there was no second aliquot to repeat the analysis and, therefore, these samples were excluded from the present study. Plasma samples were analyzed at the Clinical Neurochemistry Laboratory, University of Gothenburg (Gothenburg, Sweden) in May-June 2021.

IA-Elc

CSF and plasma samples were analyzed using the Elecsys Aβ42 and Aβ40 immunoassays on a **cobas e** 601 analyzer at the Clinical Neurochemistry Laboratory, University of Gothenburg (Gothenburg, Sweden). CSF and plasma Aβ42 and Aβ40 assays were performed as previously described.[6] For plasma assays, different calibrator range and plasma controls were used. For plasma Aβ40 analysis, a biotinylated monoclonal Aβ40 specific antibody (23C2) and a monoclonal β-Amyloid-specific antibody (3D6) labeled with a ruthenium complex were used. The Elecsys β-Amyloid(1−42) CSF immunoassay is approved for use in countries accepting the CE mark; the Elecsys β-Amyloid(1−40) CSF immunoassay is a robust prototype assay (for research use only) which is not commercially available. Plasma samples were analyzed in November-December 2017.

IA-EI

CSF and plasma Aβ42 and Aβ40 were quantified using CSF and plasma kits according to the manufacturer's instructions. Samples were analyzed at EUROIMMUN AG (Luebeck, Germany) between May and November 2017.

IA-N4PE and IA-Quan

N4PE Simoa immunoassays are specific for the first amino acid of Aβ. These assays were developed by Amsterdam UMC and ADxNeurosciences and are now commercially available from Quanterix.[7,13] Plasma samples were analyzed using N4PE 4-plex kit according to the manufacturer's instructions. Plasma levels of Aβ42 and Aβ40 were also quantified using single-plex Simoa kits from Quanterix as previously described.[8] The Simoa Aβ40 and Aβ42 assays both utilize the same capture antibody targeting the N-terminus of β-amyloid and different C-terminus detection antibodies specific to Aβ40 and Aβ42. The assays use β-amyloid (1-40) and (1-42) peptides as standards. The antibody pairs are the same in the IA-Quan Aβ42/Aβ40 single-plex kits and widely used Simoa Neuro 3-plex kit, but in the 3-plex kit the detection antibodies from the single-plex kits are used as capture antibodies and vice versa.

IA-N4PE and IA-Quan analyses were performed at the Neurochemistry laboratory of the Amsterdam UMC location VUmc (Amsterdam, Netherlands) in May-June 2020 and Quanterix Corporation (Lexington, MA, USA) between December 2014 and January 2015, respectively.

© 2021 Janelidze S et al. *JAMA Neurology.*

[^{18}F]flutemetamol PET

Cerebral Aβ deposition was visualized with the PET tracer [^{18}F]flutemetamol (approved by the Food and Drug Administration, and the European Medical Agency). [^{18}F]flutemetamol was manufactured at the radiopharmaceutical production site in Risø, Denmark, using a FASTlab synthesizer module (GE Healthcare, Cleveland, OH). Subjects received a single dose of [^{18}F]flutemetamol according to a method described previously.[14] PET/CT scanning of the brain was conducted at two sites using the same type of scanner (Gemini, Philips Healthcare, Best, the Netherlands). Sum images (from 90-110 min post injection) were analyzed using the software NeuroMarQ (GE Healthcare, Cleveland, OH, USA). [^{18}F]flutemetamol activity was quantified with a previously described fully automated PET-only method that uses an adaptive template for handling different uptake patterns in negative and positive [18F]flutemetamol images.[15] [^{18}F]flutemetamol images were spatially normalized to Montreal Neurological Institute template space using the adaptive template method. A volume of interest (VOI) template was applied for the following 9 bilateral regions: prefrontal, parietal, lateral temporal, medial temporal, sensorimotor, occipital, anterior cingulate, posterior cingulate/precuneus, and a global neocortical composite region composed by all these regions.[15] The standardized uptake value ratio (SUVR) was defined as the uptake in a VOI normalized for the cerebellar cortex uptake.

ADNI

ADNI plasma samples were analyzed between December 2020 and March 2021. Detailed information on sample handling procedures, assay protocols and performance are available from the ADNI database (for up-to-date information, see www.adni-info.org and adni.loni.usc.edu).

© 2021 Janelidze S et al. *JAMA Neurology.*

eFigure

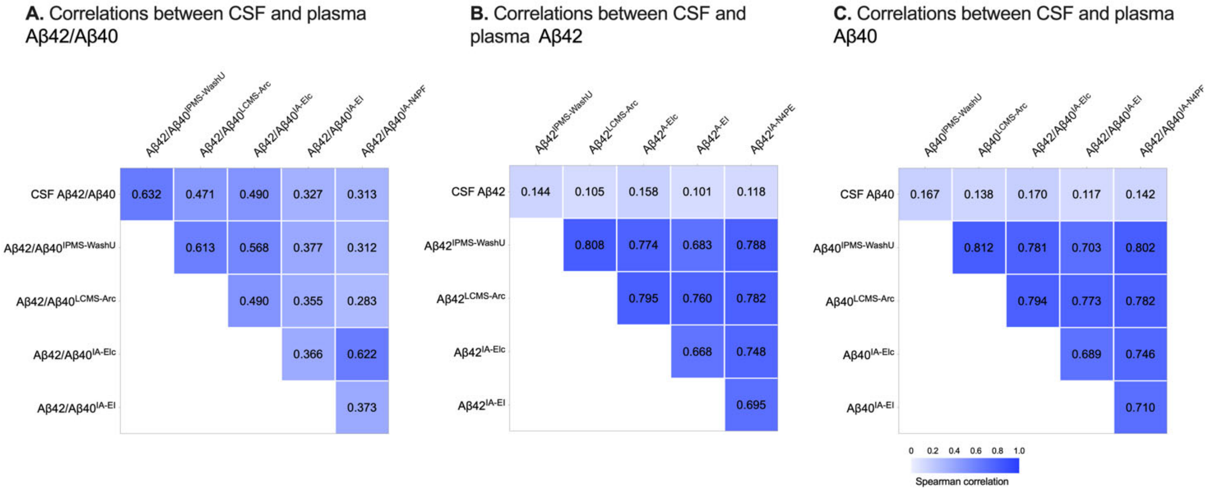

Correlations between plasma and CSF Aβ in the whole cohort (n=286). Heatmaps showing Spearman coefficients for correlations between plasma Aβ measured using IP-MS-WashU, LC-MS-Arc, IA-Elc, IA-EI and IA-N4PE and CSF Aβ measured using Elecsys immunoassays.

Aβ, amyloid-β; CSF, cerebrospinal fluid; IA-EI, immunoassay from Euroimmun; IA-Elc, Elecsys immunoassay from Roche Diagnostics; IA-N4PE, N4PE Simoa immunoassay from Quanterix assay; IPMS-WashU, immunoprecipitation-coupled mass spectrometry method developed at Washington University; LCMS-Arc, antibody-free liquid chromatography-mass spectrometry method developed by Araclon.

© 2021 Janelidze S et al. *JAMA Neurology.*

eTable 1. Plasma Aβ42 and Aβ40 assays in BioFINDER

**Assay/platform**	Capture IA antibodies	Average intra-assay CV, %	Average inter-assay CV, %	Failed samples, %
IP-MS-WashU				
Aβ42	NA	0.69	4.18	0
Aβ40	NA	0.53	5.43	0
Aβ42/Aβ40	NA	0.72	3.46	0
LC-MS-Arc				
Aβ42	NA	NA	6.0	14.6*
Aβ40	NA	NA	4.4	13.7*
IP-MS-UGOT				
Aβ42	NA	12.5	13.2	4
Aβ40	NA	5.8	7.4	4
IA-Elc				
Aβ42	Aβ(1−42)	1.2	1.9	0
Aβ40	Aβ(1−40)	1.1	1.0	0
IA-EI				
Aβ42	Aβ(1−42)	1.7	7.1	0
Aβ40	Aβ(1−40)	2.2	6.0	0
IA-N4PE				
Aβ42	Aβ(1−42)	2.3	5.0	0
Aβ40	Aβ(1−40)	2.1	5.0	0
IP-MS-Shim				
Aβ42	NA	6.9	16.7	12.3**
Aβ40	NA	7.4	11.0	12.3**
Aβ42/Aβ40	NA	5.7	10.1	12.3**
IA-Quan				
Aβ42	Aβ(N-42)	7.4	10.6	0
Aβ40	Aβ(N-40)	3.1	11.0	0

* Analysis of Aβ40 and Aβ42 failed due to an over-pressure in the chomatographic system.
** ** One batch of assay was omitted due to unexpected system unstability
Abbreviations: CV, coefficient of variability; IA, immunoassay; N/A, not available.

© 2021 Janelidze S et al. *JAMA Neurology.*

eTable 2. Preanalytical sample handling in BioFINDER

**Assay/platform**	Thaw temperature	Sample mixing	Centrifugation	Sample dilution	Sample volume (after dilution) per replicate
IP-MS-WashU	RT (22°C)	1000 rpm, 10 min	10000 rcf, 5 min	undiluted	0.45ml
LC-MS-Arc	RT	Brief vortexing	14000 rcf, 5 min	undiluted	0.2ml
IP-MS-UGOT	RT	Brief vortexing	Only in case debris was detected visually (14000 rcf, 1 min)	undiluted	0.25ml
IA-Elc	RT	Rolling mixer, 20min	Only in case debris was detected (14000 rcf, 1 min)	undiluted	Aβ42 35μl<br>Aβ40 30μl
IA-EI	RT	Brief vortexing	No	1:4	80μl
IA-N4PE	In front of a cold-air fan at RT, for ~ 15 minutes	Brief vortexing	10000 rcf, 10min	4-fold automated dilution on board of the Simoa	≥80μl provided to Simoa for two replicates (*i.e.*, 25μl*replicates+30μl dead volume)
IP-MS-Shim	RT	Brief vortexing	2273g, 5min	1:1	0.3ml
IA-Quan	RT	Brief vortexing	20000 rcf, 3min	Aβ42 1:4<br>Aβ40 1:8	0.1ml

Abbreviations: min, minutes; rcf, relative centrifugal force; rpm, evolutions per minute; RT, room temperature

© 2021 Janelidze S et al. *JAMA Neurology.*

eTable 3. Characteristics of a subcohort of participants in BioFINDER where plasma Aβ was measured using IP-MS-Shim

	**Aβ-negative n=114**	**Aβ-positive n=86**	**P-value**
Diagnosis, CU/MCI, n	80/34	33/53	8.0e-6
Age, years	69.0 (66.0-75.0)	74.0 (70.0-77.0)	0.0004
Female, n (%)	61 (53.5)	32 (37.2)	0.031
Duration of education, years [a]	12.0 (9.0-14.0)	10.0 (9.0-13.0)	0.020
MMSE	29.0 (28.0-30.0)	28.0 (26.0-29.0)	8.9e-5
APOE ε4 positivity, n (%) [a]	23 (20.4)	60 (69.8)	2.7e-12
Aβ-PET, [^{18}F]Flutemetamol SUVR	1.19 (1.12-1.26)	1.88 (1.67-2.19)	1.1e-33
CSF Aβ42/Aβ40	0.094 (0.083-0.103)	0.038 (0.033-0.048)	1.1e-33
Plasma Aβ42/Aβ40			
Aβ42/Aβ40$^{IPMS\text{-}WashU}$	0.132 (0.127-0.139)	0.122 (0.117-0.125)	2.2e-19
Aβ42/Aβ40$^{IPMS\text{-}Shim}$	0.052 (0.049-0.059)	0.046 (0.043-0.049)	3.9e-15
Aβ42/Aβ40$^{LCMS\text{-}Arc}$	0.321 (0.294-0.346)	0.285 (0.265-0.305)	3.0e-11
Aβ42/Aβ40$^{IA\text{-}Elc}$	0.067 (0.064-0.073)	0.062 (0.058-0.065)	3.9e-11
Aβ42/Aβ40$^{IA\text{-}EI}$	0.182 (0.159-0.199)	0.160 (0.145-0.174)	8.3e-7
Aβ42/Aβ40$^{IA\text{-}N4PE}$	0.133 (0.117-0.149)	0.118 (0.104-0.132)	1.5e-5

Aβ status was defined using the CSF Aβ42/Aβ40 cutoff as described in the Methods.
Data are shown as median (interquartile range) unless otherwise specified. Differences between the groups were tested using Mann-Whitney U test and Fisher's Exact test (diagnosis, sex and *APOE*).
[a] Education is missing for two study participants; *APOE* ε4 is missing for one study participant.

Aβ, amyloid-β; CSF, cerebrospinal fluid; CU, cognitively unimpaired; F, female; IA-EI, immunoassay from Euroimmun; IA-Elc, Elecsys immunoassay from Roche Diagnostics; IA-N4PE, N4PE Simoa immunoassay from Quanterix assay; IPMS-Shim, immunoprecipitation coupled mass spectrometry method developed by Shimadzu; IPMS-WashU, immunoprecipitation-coupled mass spectrometry method developed at Washington University; LCMS-Arc, antibody-free liquid chromatography-mass spectrometry method developed by Araclon; MCI, mild cognitive impairment; MMSE, Mini Mental State Examination; PET positron emission tomography.

© 2021 Janelidze S et al. *JAMA Neurology.*

eTable 4. Characteristics of a subcohort of participants in BioFINDER where plasma Aβ was measured using IP-MS-UGOT and IA-Quan

	**Aβ-negative n=139**	**Aβ-positive n=94**	**P-value**
Diagnosis, CU/MCI, n	107/29	49/42	0.0001
Age, years	71.5 (67.0-75.0)	73.0 (70.0-76.0)	0.017
Female, n (%)	78 (57.4.0)	42 (46.2)	0.105
Duration of education, years [a]	12.0 (9.0-14.0)	11.0 (9.0-13.0)	0.11
MMSE	29.0 (28.0-30.0)	28.0 (26.0-29.0)	0.0001
APOE ε4 positivity No., %	25 (18.4)	58 (63.7)	4.5e-12
Aβ-PET, [^{18}F]Flutemetamol SUVR	1.19 (1.11-1.27)	1.83 (1.54-2.12)	4.4e-30
CSF Aβ42/Aβ40	0.093 (0.079-0.102)	0.041 (0.034-0.049)	2.7e-37
Plasma Aβ42/Aβ40			
Aβ42/Aβ40$^{\text{IPMS-WashU}}$	0.132 (0.126-0.139)	0.122 (0.117-0.126)	6.3e-18
Aβ42/Aβ40$^{\text{LCMS-Arc}}$	0.322 (0.299-0.344)	0.289 (0.266-0.305)	1.8e-11
Aβ42/Aβ40$^{\text{IPMS-UGOT}}$	0.073 (0.063-0.094)	0.060 (0.048-0.077)	6.0e-6
Aβ42/Aβ40$^{\text{IA-Elc}}$	0.067 (0.065-0.072)	0.062 (0.057-0.065)	4.7e-14
Aβ42/Aβ40$^{\text{IA-EI}}$	0.183 (0.165-0.200)	0.165 (0.148-0.175)	4.6e-7
Aβ42/Aβ40$^{\text{IA-N4PE}}$	0.136 (0.121-0.148)	0.119 (0.107-0.133)	1.4e-7
Aβ42/Aβ40$^{\text{IA-Quan}}$	0.069 (0.061-0.080)	0.063 (0.056-0.074)	0.001

Aβ status was defined using the CSF Aβ42/Aβ40 cutoff as described in the Methods.
Data are shown as median (interquartile range) unless otherwise specified. Differences between the groups were tested using Mann-Whitney U test and Fisher's Exact test (diagnosis, sex and *APOE*).
[a] Education is missing for one study participant.

Aβ, amyloid-β; CSF, cerebrospinal fluid; CU, cognitively unimpaired; F, female; IA-EI, immunoassay from Euroimmun; IA-Elc, Elecsys immunoassay from Roche Diagnostics; IA-N4PE, N4PE Simoa immunoassay from Quanterix assay; IA-Quan, Simoa immunoassay from Quanterix; IPMS-UGOT, immunoprecipitation-coupled mass spectrometry method developed at the University of Gothenburg; IPMS-WashU, immunoprecipitation-coupled mass spectrometry method developed at Washington University; LCMS-Arc, antibody-free liquid chromatography-mass spectrometry method developed by Araclon; MCI, mild cognitive impairment; MMSE, Mini Mental State Examination; PET positron emission tomography.

© 2021 Janelidze S et al. *JAMA Neurology.*

eTable 5. Plasma concentrations of Aβ42 and Aβ40 in BioFINDER

	**Aβ-negative n=168**	**Aβ-positive n=118**	**P-value**
$A\beta42^{IPMS\text{-}WashU}$	33.1 (30.2-36.0)	30.3 (27.3-33.5)	1.0e-5
$A\beta40^{IPMS\text{-}WashU}$	250.8 (230.3-279.2)	247.4 (226.3-279.2)	0.73
$A\beta42^{LCMS\text{-}Arc}$	92.4 (81.8-99.9)	79.9 (72.4-94.3)	1.3e-7
$A\beta40^{LCMS\text{-}Arc}$	279.8 (261.5-303.8)	284.9 (251.3-316.0)	0.79
$A\beta42^{IA\text{-}Elc}$	31.9 (29.4-34.3)	29.3 (26.8-32.8)	1.8e-5
$A\beta40^{IA\text{-}Elc}$	469.0 (438.3-505.8)	476.0 (435.8-529.0)	0.24
$A\beta42^{IA\text{-}EI}$	27.6 (22.7-31.3)	25.3 (20.9-28.9)	0.007
$A\beta40^{IA\text{-}EI}$	150.4 (138.7-167.1)	155.6 (136.7-177.9)	0.20
$A\beta42^{IA\text{-}N4PE}$	11.5 (9.8-13.1)	10.1 (8.7-11.9)	0.0005
$A\beta40^{IA\text{-}N4PE}$	85.0 (76.2-98.0)	87.8 (73.9-98.6)	0.70
$A\beta42^{IPMS\text{-}Shim}$	0.488 (0.426-0.572) n=114	0.454 (0.383-0-528) n=86	0.005
$A\beta40^{IPMS\text{-}Shim}$	9.1 (8.2-10.1) n=114	9.5 (8.2-11.2) n=86	0.10
$A\beta42^{IPMS\text{-}UGOT}$	17.4 (14.2-22.5) n=136	14.8 (11.7-17.8) n=91	0.0001
$A\beta40^{IPMS\text{-}UGOT}$	230.5 (209.0-264.0) n=136	234.3 (210.8-265.4) n=91	0.45
$A\beta42^{IA\text{-}Quan}$	19.1 (16.5-22.7) n=136	17.4 (14.8-20.8) n=91	0.011
$A\beta40^{IA\text{-}Quan}$	269.3 (238.4-307.3) n=136	270.7 (231.1-323.2) n=91	0.84

Aβ status was defined using the CSF Aβ42/Aβ40 cutoff as described in the Methods.
Biomarker concentrations are shown in pg/ml except $A\beta42^{IPMS\text{-}Shim}$ and $A\beta40^{IPMS\text{-}Shim}$ where the values are signal intensity normalized to the internal standard SIL-Aβ(1–38).

Aβ, amyloid-β; IA-EI, immunoassay from Euroimmun; IA-Elc, Elecsys immunoassay from Roche Diagnostics; IA-N4PE, N4PE Simoa immunoassay from Quanterix; IPMS-UGOT, immunoprecipitation-coupled mass spectrometry method developed at the University of Gothenburg; IA-Quan, Simoa immunoassay from Quanterix; IPMS-Shim, immunoprecipitation coupled mass spectrometry method developed by Shimadzu; IPMS-WashU, immunoprecipitation-coupled mass spectrometry method developed at Washington University; LCMS-Arc, antibody-free liquid chromatography-mass spectrometry method developed by Araclon.

© 2021 Janelidze S et al. *JAMA Neurology.*

eTable 6. ROC analysis for abnormal CSF Aβ42 and Aβ40^{EI} status in BioFINDER

	**CSF Aβ42/Aβ40**
**Whole cohort, Aβ+/Aβ-, n**	118/168
Aβ42/Aβ40$^{IPMS\text{-}WashU}$	0.852 [0.807-0.896]
Aβ42/Aβ40$^{IA\text{-}Elc}$	0.773 [0.719-0.827] [a]
Aβ42/Aβ40$^{LCMS\text{-}Arc}$	0.761 [0.704-0.818] [a]
Aβ42/Aβ40$^{IA\text{-}EI}$	0.688 [0.626-0.750] [b]
Aβ42/Aβ40$^{IA\text{-}N4PE}$	0.670 [0.608-0.732] [b]
**Sub-cohort with Aβ42/Aβ40$^{IPMS\text{-}Shim}$, Aβ+/Aβ-, n**	86/114
Aβ42/Aβ40$^{IPMS\text{-}WashU}$	0.864 [0.814-0.914]
Aβ42/Aβ40$^{IPMS\text{-}Shim}$	0.834 [0.779-0.890]
Aβ42/Aβ40$^{LCMS\text{-}Arc}$	0.783 [0.720-0.847] [c]
Aβ42/Aβ40$^{IA\text{-}Elc}$	0.773 [0.709-0.837] [a]
Aβ42/Aβ40$^{IA\text{-}EI}$	0.720 [0.648-0.791] [a]
Aβ42/Aβ40$^{IA\text{-}N4PE}$	0.668 [0.594-0.743] [b]
**Sub-cohort with Aβ42/Aβ40$^{IA\text{-}Quan}$, Aβ+/Aβ-, n**	95/138
Aβ42/Aβ40$^{IPMS\text{-}WashU}$	0.842 [0.791-0.894]
Aβ42/Aβ40$^{IA\text{-}Elc}$	0.797 [0.740-0.854]
Aβ42/Aβ40$^{LCMS\text{-}Arc}$	0.751 [0.684-0.817] [a]
Aβ42/Aβ40$^{IPMS\text{-}UGOT}$	0.710 [0.640-0.780] [a]
Aβ42/Aβ40$^{IA\text{-}EI}$	0.692 [0.622-0.763] [b]
Aβ42/Aβ40$^{IA\text{-}N4PE}$	0.691 [0.622-0.759] [b]
Aβ42/Aβ40$^{IA\text{-}Quan}$	0.633 [0.559-0.706] [b]

Data are shown as AUC (95% CI); AUC of two ROC curves were compared with DeLong test.
Out of 322 participants, 185 were classified as CSF Aβ42/Aβ40 negative and 137 were classified as CSF Aβ42/Aβ40 positive. CSF Aβ42/Aβ40 data was binarized using previously described cutoff (0.091).[16]

[a] $p<0.01$; [b] $p<0.001$; [c] $p<0.05$ compared with Aβ42/Aβ40$^{IPMS\text{-}WashU}$

Aβ, amyloid-β; AUC, area under the curve; CSF, cerebrospinal fluid; IA-EI, immunoassay from Euroimmun; IA-Elc, Elecsys immunoassay from Roche Diagnostics; IA-N4PE, N4PE Simoa immunoassay from Quanterix; IA-Quan, Simoa immunoassay from Quanterix; IPMS-Shim, immunoprecipitation coupled mass spectrometry method developed by Shimadzu; IPMS-WashU, immunoprecipitation-coupled mass spectrometry method developed at Washington University; IPMS-WashU, immunoprecipitation-coupled mass spectrometry method developed at the University of Gothenburg; LCMS-Arc, antibody-free liquid chromatography-mass spectrometry method developed by Araclon; ROC, receiver operating characteristic.

© 2021 Janelidze S et al. *JAMA Neurology.*

eTable 7. ROC analysis of plasma Aβ42 and Aβ40 combined with A*POE* ε4 genotype for abnormal CSF Aβ42 and Aβ40 in BioFINDER

**Plasma Aβ42/Aβ40 + A*POE* ε4**	**CSF Aβ42/Aβ40**
**Whole cohort, Aβ+/Aβ-, n**	118/167
Aβ42/Aβ40$^{IPMS-WashU}$	0.882 [0.842-0.922]
Aβ42/Aβ40$^{LCMS-Arc}$	0.841 [0.794-0.887] [a]
Aβ42/Aβ40^{IA-Elc}	0.820 [0.771-0.869] [a]
Aβ42/Aβ40^{IA-EI}	0.794 [0.741-0.846] [b]
Aβ42/Aβ40$^{IA-N4PE}$	0.783 [0.729-0.836] [b]
**Sub-cohort with Aβ42/Aβ40$^{IPMS-Shim}$, Aβ+/Aβ-, n**	86/113
Aβ42/Aβ40$^{IPMS-WashU}$	0.902 [0.861-0.944]
Aβ42/Aβ40$^{IPMS-Shim}$	0.868 [0.819-0.918]
Aβ42/Aβ40$^{LCMS-Arc}$	0.863 [0.812-0.913]
Aβ42/Aβ40^{IA-Elc}	0.834 [0.778-0.889] [b]
Aβ42/Aβ40^{IA-EI}	0.816 [0.757-0.875] [b]
Aβ42/Aβ40$^{IA-N4PE}$	0.798 [0.736-0.861] [b]
**Sub-cohort with Aβ42/Aβ40$^{IPMS-UGOT}$ and Aβ42/Aβ40$^{IA-Quan}$, Aβ+/Aβ-, n**	91/136
Aβ42/Aβ40$^{IPMS-WashU}$	0.870 [0.823-0.917]
Aβ42/Aβ40$^{LCMS-Arc}$	0.841 [0.788-0.894]
Aβ42/Aβ40^{IA-Elc}	0.841 [0.790-0.891]
Aβ42/Aβ40$^{IPMS-UGOT}$	0.805 [0.747-0.864] [a]
Aβ42/Aβ40^{IA-EI}	0.805 [0.747-0.864] [a]
Aβ42/Aβ40$^{IA-N4PE}$	0.794 [0.735-0.854] [a]
Aβ42/Aβ40$^{IA-Quan}$	0.779 [0.717-0.841] [b]

Data are shown as AUC (95% CI); AUC of two ROC curves were compared with DeLong test.
CSF Aβ42/Aβ40 data was binarized using previously described cutoff (0.059).[6]

[a] $p<0.05$; [b] $p<0.01$ compared with Aβ42/Aβ40$^{IPMS-WashU}$

Aβ, amyloid-β; AUC, area under the curve; CSF, cerebrospinal fluid; IA-EI, immunoassay from Euroimmun; IA-Elc, Elecsys immunoassay from Roche Diagnostics; IA-N4PE, N4PE Simoa immunoassay from Quanterix; IA-Quan, Simoa immunoassay from Quanterix; IPMS-Shim, immunoprecipitation coupled mass spectrometry method developed by Shimadzu; IPMS-WashU, immunoprecipitation-coupled mass spectrometry method developed at Washington University; LCMS-Arc, antibody-free liquid chromatography-mass spectrometry method developed by Araclon; ROC, receiver operating characteristic; PET positron emission tomography.

eTable 8. Plasma concentrations of Aβ42 and Aβ40 in ADNI

	**Aβ-negative n=63**	**Aβ-positive n=59**	**P-value**
$A\beta42^{IPMS\text{-}WashU}$	37.0 (34.8-42.3)	34.5 (31.3-40.0)	0.005
$A\beta40^{IPMS\text{-}WashU}$	281.2 (263.0-312.2)	279.4 (260.2-325.4)	0.73
$A\beta42^{IPMS\text{-}Shim}$	0.324 (0.290-0.363)	0.293 (0.266-0.343)	0.004
$A\beta40^{IPMS\text{-}Shim}$	8.0 (7.0-8.9)	8.0 (6.7-9.6)	0.75
$A\beta42^{IPMS\text{-}UGOT}$	19.7 (17.3-25.1)	17.2 (13.2-21.7)	0.013
$A\beta40^{IPMS\text{-}UGOT}$	277.0 (252.0-313.0)	273.0 (236.0-322.0)	0.49
$A\beta42^{IA\text{-}Elc}$	52.7 (48.0-60.8)	47.0 (42.0-54.6)	0.003
$A\beta40^{IA\text{-}Elc}$	316.0 (287.0-339.0)	313.0 (278.0-355.0)	0.87
$A\beta42^{IA\text{-}N4PE}$	7.8 (6.8-8.9)	7.1 (6.2-8.4)	0.036
$A\beta40^{IA\text{-}N4PE}$	164.1 (134.2-194.6)	160.7 (133.9-208.8)	0.65
$A\beta42^{IA\text{-}Quan}$	13.8 (12.0-15.4)	13.0 (11.7-14.8)	0.24
$A\beta40^{IA\text{-}Quan}$	341.0 (305.0-374.0)	353.0 (303.0-400.0)	0.43

Aβ status was defined using previously described Aβ-PET cutoff (1.11).[17,18]. Biomarker concentrations are shown in pg/ml except $A\beta42^{IPMS\text{-}Shim}$ and $A\beta40^{IPMS\text{-}Shim}$ where the values are signal intensity normalized to the internal standard SIL-Aβ(1–38).

Aβ, amyloid-β; IA-Elc, Elecsys immunoassay from Roche Diagnostics; IA-N4PE, N4PE Simoa immunoassay from Quanterix; IA-Quan, Simoa immunoassay from Quanterix; IPMS-UGOT, immunoprecipitation-coupled mass spectrometry method developed at the University of Gothenburg; IPMS-Shim, immunoprecipitation coupled mass spectrometry method developed by Shimadzu; IPMS-WashU, immunoprecipitation-coupled mass spectrometry method developed at Washington University.

© 2021 Janelidze S et al. *JAMA Neurology.*

eReferences

1. Petersen RC. Mild cognitive impairment as a diagnostic entity. *J Intern Med.* 2004;256(3):183-194.
2. Mattsson N, Insel PS, Palmqvist S, et al. Increased amyloidogenic APP processing in APOE varepsilon4-negative individuals with cerebral beta-amyloidosis. *Nat Commun.* 2016;7:10918.
3. Jack CR, Jr., Bennett DA, Blennow K, et al. NIA-AA Research Framework: Toward a biological definition of Alzheimer's disease. *Alzheimers Dement.* 2018;14(4):535-562.
4. Ovod V, Ramsey KN, Mawuenyega KG, et al. Amyloid beta concentrations and stable isotope labeling kinetics of human plasma specific to central nervous system amyloidosis. *Alzheimers Dement.* 2017;13(8):841-849.
5. Palmqvist S, Janelidze S, Quiroz YT, et al. Discriminative Accuracy of Plasma Phospho-tau217 for Alzheimer Disease vs Other Neurodegenerative Disorders. *JAMA.* 2020.
6. Palmqvist S, Janelidze S, Stomrud E, et al. Performance of Fully Automated Plasma Assays as Screening Tests for Alzheimer Disease-Related beta-Amyloid Status. *JAMA Neurol.* 2019.
7. Verberk IMW, Slot RE, Verfaillie SCJ, et al. Plasma Amyloid as Prescreener for the Earliest Alzheimer Pathological Changes. *Ann Neurol.* 2018;84(5):648-658.
8. Janelidze S, Stomrud E, Palmqvist S, et al. Plasma beta-amyloid in Alzheimer's disease and vascular disease. *Sci Rep.* 2016;6:26801.
9. Nakamura A, Kaneko N, Villemagne VL, et al. High performance plasma amyloid-beta biomarkers for Alzheimer's disease. *Nature.* 2018;554(7691):249-254.
10. Schindler SE, Bollinger JG, Ovod V, et al. High-precision plasma beta-amyloid 42/40 predicts current and future brain amyloidosis. *Neurology.* 2019;93(17):e1647-e1659.
11. Janelidze S, Palmqvist S, Leuzy A, et al. Detecting amyloid positivity in early Alzheimer's disease using combinations of plasma Aβ42/Aβ40 and P-tau. *Alzheimer's & Dementia.* 2021;In press.
12. Keshavan A, Pannee J, Karikari TK, et al. Population-based blood screening for preclinical Alzheimer's disease in a British birth cohort at age 70. *Brain.* 2021;144(2):434-449.
13. Thijssen EH, Verberk IMW, Vanbrabant J, et al. Highly specific and ultrasensitive plasma test detects Abeta(1-42) and Abeta(1-40) in Alzheimer's disease. *Sci Rep.* 2021;11(1):9736.
14. Koole M, Lewis DM, Buckley C, et al. Whole-body biodistribution and radiation dosimetry of 18F-GE067: a radioligand for in vivo brain amyloid imaging. *J Nucl Med.* 2009;50(5):818-822.
15. Lundqvist R, Lilja J, Thomas BA, et al. Implementation and validation of an adaptive template registration method for 18F-flutemetamol imaging data. *J Nucl Med.* 2013;54(8):1472-1478.
16. Minta K, Brinkmalm G, Janelidze S, et al. Quantification of total apolipoprotein E and its isoforms in cerebrospinal fluid from patients with neurodegenerative diseases. *Alzheimers Res Ther.* 2020;12(1):19.
17. Landau SM, Lu M, Joshi AD, et al. Comparing positron emission tomography imaging and cerebrospinal fluid measurements of beta-amyloid. *Ann Neurol.* 2013;74(6):826-836.
18. Landau SM, Mintun MA, Joshi AD, et al. Amyloid deposition, hypometabolism, and longitudinal cognitive decline. *Ann Neurol.* 2012;72(4):578-586.

eAppendix. ADNI Investigators

Michael Weiner
Paul Aisen
Ronald Petersen
Clifford R. Jack Jr.
William Jagust
John Q. Trojanowski
Arthur W. Toga
Laurel Beckett
Robert C. Green
Andrew J. Saykin
John Morris
Leslie M. Shaw
Enchi Liu
Tom Montine
Ronald G. Thomas
Michael Donahue
Sarah Walter
Devon Gessert
Tamie Sather
Gus Jiminez
Danielle Harvey
Natthew Bernstein
Nick Fox
Paul Thompson
Norbert Schuff
Charles DeCarli
Bret Borowski
Jeff Gunter
Matt Senjem
Prashanti Vemuri
David Jones
Kejal Kantarci
Chard Ward
Robert A. Koeppe
Norm Foster
Eric M. Reiman
Kewei Chen
Chet Mathis
Susan Landau
Nigel J. Cairns
Erin Householder
Lisa T. Reinwald
Virginia Lee
Magdalena Korecka
Michal Figurski
Karen Crawford
Scott Neu
Tatiana M. Foroud
Steven Potkin
Li Chen
Faber Kelley
Sungeun Kim
Kwangsik Nho
Zaven Kachaturian
Richard Frank

© 2021 Janelidze S et al. *JAMA Neurology.*

Peter J. Snyder
Susan Molchan
Jeffrey Kaye
Joseph Quinn
Betty Lind
Raina Carter
Sara Dolen
Lon S. Schneider
Sonia Pawluczyk
Mauricio Beccera
Liberty Teodoro
Bryan M. Spann
James Brewer
Helen Vanderswag
Adam Fleisher
Judith L. Heidebrink
Joanne L. Ford
Sara S. Mason
Colleen S. Albers
David Knopman
Kris Johnson
Rachelle S. Doody
Javier Villanueva
Munir Chowdhury
Susan Rountree
Mimi Dang
Yaakov Stern
Lawrence S. Honig
Karen L. Bell
Beau Ances
Maria Carroll
Sue Leon
Erin Householder
Mark A. Mintun
Stacy Schneider
Angela Oliver
Randal Griffith
David Clark
David Geldmacher
John Brockington
Erik Roberson
Hillel Grossman
Effie Mitsis
Leyla deToledo-Morrell
Raj C. Shah
Ranjan Duara
Daniel Varon
Maria T. Greig
Peggy Roberts
Marilyn Albert
Chiadi Onyike
Daniel D'Agostino
Stephanie Kleib
James E. Galvin
Dana M. Pogorelec
Brittany Cerbone
Christina A. Michel

© 2021 Janelidze S et al. *JAMA Neurology.*

Henry Rusinek
Mony J. De Leon
Lidia Glodzik
Susan De Santi
Murali Doraiswamy
Jeffrey R. Petrella
Terence Z. Wong
Jason H. Karlawish
David Wolk
Charles D. Smith
Greg Jicha
Peter Hardy
Partha Sinha
Elizabeth Oates
Gary Conrad
Oscar L. Lopez
MaryAnn Oakley
Donna M. Simpson
Anton P. Porsteinsson
Bonnie S. Goldstein
Kim Martin
Kelly M. Makino
M. Saleem Ismael
Connie Brand
Ruth A. Mulnard
Gaby Thai
Catherina McAdams Ortiz
Kyle Womack
Dana M. Mathews
Mary Quiceno
Ramon Diaz Arrastia
Richard King
Myron Steiner
Kristen Martin Cook
Michael Devous
Allan I. Levey
James J. Lah
Janet S. Cellar
Jeffrey M. Burns
Heather S. Anderson
Russell h. Swerdlov
Liana Apostolova
Kathleen Tingus
Ellen Woo
Daniel H. Silverman
Po H. Lu
George Bartzokis
Neill R. Graff-Radford
Francine Parfitt
Tracey Kendall
Heather S. Johnson
Martin R. Farlow
Ann-Marie Hake
Brandy R. Matthews
Scott Herring
Cynthia Hunt
Christopher H. van Dyck

© 2021 Janelidze S et al. *JAMA Neurology.*

Richard E. Carson
Martyha G. MacAvoy
Howard Chertkow
Howard Bergman
Chris Hosein
Sandra Black
Bojana Stefanovic
Curtis Caldwell
Ging Yuek Robin
Howard Feldman
Benita Mudge
Michele Assaly Past
Andrew Kertesz
John Rogers
Dick Trost
Charles Bernick
Donna Munic
Diana Kerwin
Marek M. Mesulam
Kristine Lipowski
Chuang Kuo Wu
Nancy Johnson
Carl Sadowsky
Walter Martinez
Raymond S. Turner
Kathleen Johnson
Brigid Reynolds
Reisa A. Sperling
Keith A. Johnson
Gad Marshall
Meghan Frey
Jerome Yesavage
Joy L. Taylor
Barton Lane
Allyson Rosen
Jared Tinklenberg
Marwan N. Sabbagh
Christine M. Belden
Sherye A. Sirrel
Neil Kowal
Ronald Killiany
Andrew E. Budson
Alexander Norbash
Patricia L. Johnson
Thomas O. Obisesan
Saba Wolday
Joana Allard
Alan Lerner
Paula Ogrocki
Leon Hudson
Evan Fletcher
Owen Carmichel
John Olichney
Smita Kittur
Michael Borrie
T.Y. Lee
Rob Bartha

© 2021 Janelidze S et al. *JAMA Neurology.*

Sterling Johnson
Sanjay Asthana
Cynthia M. Carlsson
Steven G. Potkin
Adrian Preda
Daan Nguyen
Pierre Tarlot
Stephanie Reeder
Vernice Bates
Horacio Capote
Michelle Rainka
Douglas W. Scharre
Maria Kataki
Anahita Adeli
Earl A. Zimmerman
Dzintra Celmins
Alice D. Brown
Godfrey D. Pearlson
Karen Blank
Karen Anderson
Robert B. Santulli
Tamar J. Kitzmiller
Eben S, Schwartz
Kaycee M. Sinks
Jeff D. Williamson
Pradeep Garg
Franklin Watkins
Brian R. Ott
Henry Querfurth
Geoffry Tremont
Stephen Salloway
Paul Malloy
Stephen Correia
Howard J. Rosen
Bruce L. Miller
Jacob Mintzer
Kenneth Spicer
David Bachman
Elizabether Pasternak
Irina Rachiksky
John Rogers
Andrew Kertesz
Nunzio Pomara
Raymundo Hernando
Antero Sarrael
Susan K. Schultz
Laura L. Boles
Hyungsub Shim
Karen E. Smith
Norman Relkin
Gloria Chaing
Amanda Smith
Kristin. Fargher
Balebail A. Raj

© 2021 Janelidze S et al. *JAMA Neurology.*

© 2021 Janelidze S et al. *JAMA Neurology*.
